# Effect of Moisture on the Fatigue and Self-Healing Properties of SiO_2_/SBS Composite Modified Asphalt

**DOI:** 10.3390/ma17184526

**Published:** 2024-09-14

**Authors:** Juzhong Wang, Shangjun Yu, Yihan Wang, Linhao Sun, Ruixia Li, Jinchao Yue

**Affiliations:** 1 Zhengzhou Public Utility Investment and Development Group Co., Ltd., Zhengzhou 450001, China; wangjuzhong2024@163.com; 2 School of Water Conservancy and Transportation, Zhengzhou University, Zhengzhou 450001, China; 15837119118@163.com (S.Y.); 18862973613@163.com (Y.W.); yuejc@zzu.edu.cn (J.Y.)

**Keywords:** SBS modifier, nano-SiO_2_, moisture, simplified viscoelastic continuum damage theory, fatigue and self-healing performance

## Abstract

Moisture accelerates the degradation of asphalt properties, significantly impacting the service life of roads. Therefore, this study uses simplified viscoelastic continuous damage theory and employs frequency scanning, linear amplitude scanning, and fatigue–healing–fatigue tests with a dynamic shear rheometer. The objective is to investigate the effects of aging time, moisture conditions, and aging temperature on the fatigue and self-healing performance of SBS (Styrene–Butadiene–Styrene block copolymer)-modified asphalt, nano-SiO_2_-modified asphalt, and nano-SiO_2_/SBS composite modified asphalt in a moisture-rich environment. The results indicate that nano-SiO_2_ powder enhances the low-temperature performance of modified asphalt, whereas the SBS modifier reduces temperature sensitivity and increases the recovery percentage after deformation. Compared to SBS-modified asphalt, the deformation resistance of nano-SiO_2_/SBS composite modified asphalt has increased by about 30%, while nano-SiO_2_-modified asphalt shows relatively poor deformation resistance. The fatigue performance of SBS-modified asphalt deteriorates under moisture, whereas the addition of nano-SiO_2_ powder improves its fatigue life. Nano-SiO_2_/SBS composite modified asphalt exhibits strong self-healing capabilities. Although self-healing can enhance the fatigue life of modified asphalt, moisture inhibits this improvement after self-healing.

## 1. Introduction

Asphalt pavement is continuously exposed to sunlight, temperature fluctuations, oxygen, water vapor, and other environmental factors. Concurrently, repeated vehicle loading leads to fatigue cracking. Fatigue cracking is a crucial issue in the construction and maintenance of roads, directly affecting the lifespan of asphalt pavements. How to prevent or slow down the development of cracks, thereby extending the service life of asphalt roads, has received significant attention. However, the self-healing properties of asphalt materials can enhance durability by filling these cracks during periods without load. Therefore, researchers frequently examine the role of self-healing behavior when studying the fatigue performance of asphalt materials. Laboratories often use continuous and repeated loading methods to test the fatigue performance of asphalt materials, which is different from the actual stressing mode during the service process of pavements and ignores the influence of load gap self-healing behavior on fatigue life, resulting in a lower prediction value of fatigue life for asphalt materials. Since its discovery, the self-healing behavior of asphalt materials has become a focal point of research both domestically and internationally. Scholars have conducted extensive research on the fatigue life considering this self-healing behavior. Currently, both domestic and international researchers focus on thermo-oxidative and photo-oxidative aging when studying the impact of external environments on the fatigue and self-healing performance of asphalt materials. However, in actual road use, the pavement design in the initial stage has the problem of unreasonable planning, and the aging of asphalt will affect the durability of asphalt pavement to varying degrees. Water exposure is a critical factor that accelerates the aging process of asphalt, a factor often overlooked by scholars [1,2].

In 1961, Tyaxler identified 15 causes of asphalt aging, summarizing the impact of water exposure along with factors such as time, heat, oxygen, and sunlight. He suggested that water’s effects are most significant on the road surface [3,4]. Lv Weimin [5] stated that rainwater washes away soluble substances in asphalt, altering its composition and accelerating the material’s aging and deterioration. Chen et al. [6] explored the impact of water aging temperature, humidity, and duration on the fatigue properties of asphalt materials using the simplified visco-elastic continuum model. The results indicated that increased temperature, humidity, and exposure time significantly exacerbate asphalt damage, leading to a substantial reduction in its fatigue life. Mannan et al. [7] studied the self-healing behavior of asphalt binder under moisture conditioning and found that it primarily reduces immediate healing by decreasing binder cohesion, thus lowering overall fatigue damage healing performance. Xuemei Zhang [8] analyzed the effect of water erosion on the rheological properties of 70# asphalt (70 base asphalt) and SBS-modified asphalt commonly used in Chinese road projects using a dynamic shear rheometer (DSR). The results demonstrated that SBS-modified asphalt has superior resistance to water erosion compared to 70# petroleum asphalt.

SBS-modified asphalt is widely used in China’s road paving due to its beneficial properties. However, during usage, SBS modifiers are prone to thermal decomposition and segregation, which can prevent the pavement from achieving the expected performance in service. Research has shown that the appropriate combination of nanomaterials and SBS modifiers can enhance the rheological properties of asphalt and improve high-temperature stability, rutting resistance, fatigue resistance, and waterproofing. This makes it a top choice for creating high-performance asphalt materials [9,10,11]. Huang et al. [12] examined the microstructure of nano-SiO_2_ and SBS composites using scanning electron microscopy and atomic force microscopy. They found that nano-SiO_2_ particles enhanced the interaction between SBS particles, resulting in an increased honeycomb structure in SBS-modified asphalt. Shafabakhsh et al. [10] studied the fatigue performance of asphalt modified with a composite of nano-SiO_2_ powder and SBS modifier. Their results indicated a significant improvement in the fatigue life of the modified asphalt. Rezaei et al. [13] analyzed the composite modulus, phase angle, energy storage modulus, and loss modulus of nano-SiO_2_ and SBS-modified asphalt in a medium temperature environment. They found that adding nano-SiO_2_ powder to SBS-modified asphalt enhances the fatigue resistance and medium temperature performance of the asphalt samples.

Previous studies have shown that moisture intrusion accelerates the deterioration of asphalt material properties. However, incorporating nano-SiO_2_ powder can enhance the properties of SBS-modified asphalt. Despite these findings, there is limited research on the effects of moisture on the fatigue and self-healing properties of nano-SiO_2_/SBS composite modified asphalt. Therefore, this study investigates the fatigue and self-healing performance of SBS-modified asphalt, nano-SiO_2_-modified asphalt, and nano-SiO_2_/SBS composite modified asphalt under three moisture-related conditions in the natural environment: aging time, dry and wet cycles, and aging temperature. The water-containing environment test program was designed to analyze the effects of moisture on the fatigue and self-healing behavior of these asphalt materials. The findings aim to provide a theoretical reference and scientific basis for selecting asphalt materials in road engineering.

## 2. Materials and Methods

### 2.1. Materials

**Figure 1:**
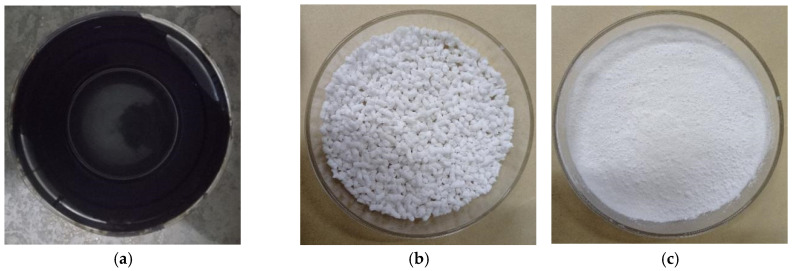
Base asphalt and modifier. (**a**) 70# base asphalt. (**b**) SBS modifier. (**c**) Nano-SiO_2_ powder.

This study selects 70# road petroleum asphalt, commonly used in China’s road projects, as the base asphalt, as illustrated in Figure 1a. The basic performance indicators of the asphalt material were tested according to the “Standard Test Methods for Asphalt and Asphalt Mixtures in Highway Engineering” (JTG E20-2011) [14]. The test results, presented in Table 1, meet the specification requirements. A mass fraction of 4% SBS modifier was selected to prepare SBS-modified asphalt, as shown in Figure 1b. The main technical parameters of the SBS modifier are listed in Table 2. Additionally, a mass fraction of 2% lipophilic hydrophobic nano-SiO_2_ powder was used to prepare nano-SiO_2_ modified asphalt, as shown in Figure 1c. The main technical parameters of the nano-SiO_2_ powder are detailed in Table 3. The base asphalt and modifiers are depicted in Figure 1.

### 2.2. Moisture Action Test Program

This study examines the effects of three water environment factors: aging time, dry and wet conditions, and aging temperature. The modified asphalt undergoes short-term aging following the AASHTO T240 standard [15], after which it is subjected to water exposure tests. The asphalt pressure aging vessel is utilized for this process. The specific operational procedure is detailed below.

(1) Aging Time

Weigh 50 ± 0.5 g of the short-term aged asphalt sample into a tray. Evenly spray 10 mL of deionized water (DW) on the asphalt surface. Place the tray on the aging shelf and insert it into the asphalt pressure aging tester. Maintain the temperature at 90 °C and an air pressure of 2.1 MPa ± 0.1 MPa. The aging process is conducted for periods of 0 h, 10 h, 20 h, and 30 h.

(2) Dry–Wet Conditions

The wet–dry cycle was selected to simulate the effect of annual rainy days on asphalt materials in various geographical regions. The water content time share was defined as the ratio of annual rainy days to total days in a year. In this experiment, 10 mL of DW was uniformly sprayed onto the surface of the asphalt specimen in the tray. The tray was then placed into the asphalt pressure aging test chamber at 90 °C, under an air pressure of 2.1 MPa ± 0.1 MPa, for durations of 0 h, 2 h, 4 h, 6 h, and 10 h. After these wet periods, the tray was removed, the surface water was drained, and the specimen was returned to the chamber for dry aging for 10 h, 8 h, 6 h, 4 h, and 0 h. This cycle was repeated three times to simulate varying water content conditions, with the water content time representing 0%, 20%, 40%, 60%, and 100% of the cycle duration. Specimens with 0% water content were used as controls to represent dry conditions.

(3) Aging Temperature

The temperature of the asphalt pressure aging test chamber was set to 30 °C, 45 °C, 60 °C, 75 °C, and 90 °C. Then, 10 mL of deionized water (DW) was uniformly sprayed onto the surface of the asphalt specimens in the trays. The trays were then placed into the asphalt pressure aging test chamber and aged for 30 h under an air pressure of 2.1 MPa ± 0.1 MPa.

The asphalt sample identifiers are summarized in Table 4. Here, ‘S’ represents SBS-modified asphalt, ‘NS’ denotes nano-SiO_2_-modified asphalt, and ‘NS/S’ stands for nano-SiO_2_/SBS composite modified asphalt. It should be noted that for the sample S/NS/(NS/S)0-100-90, an aging time of 0 h signifies that only a short-term aging treatment was performed. Additionally, the water content time percentage for the sample S/NS/(NS/S)30-0-90-DW is 0%, indicating that the specimen was maintained under dry conditions.

### 2.3. Laboratory Testing

**Figure 2:**
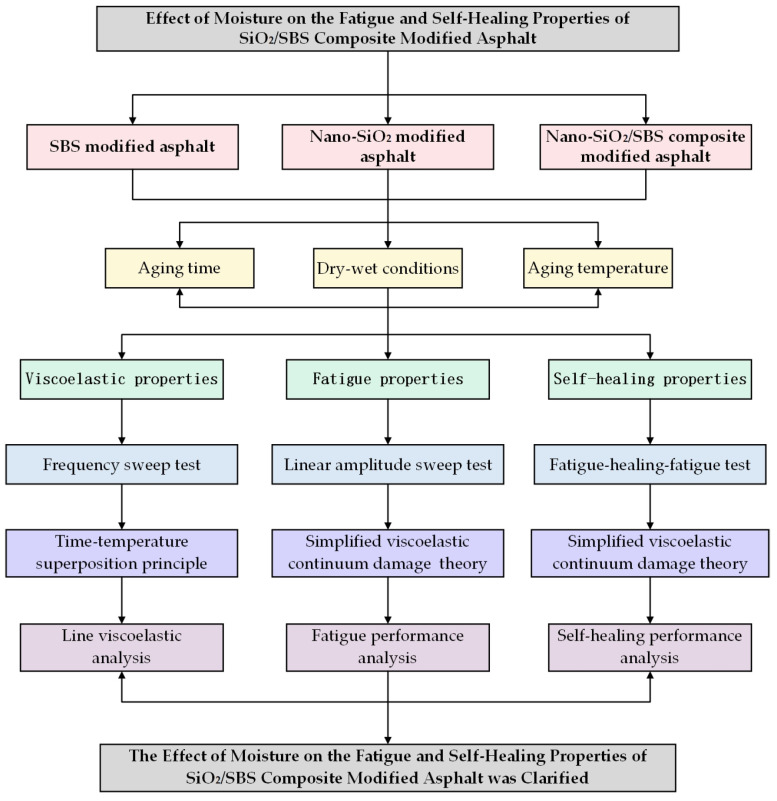
Flowchart. This is the specific test technology roadmap of this article.

Figure 2 is a flowchart.

#### 2.3.1. Frequency Sweep (FS) Test

Frequency sweep tests were conducted using a dynamic shear rheometer at a constant strain amplitude of 0.1%. The test temperatures were set at five levels: 15 °C, 20 °C, 25 °C, 30 °C, and 35 °C. The tests were conducted within a frequency range of 0.1 to 100 rad/s to obtain the linear viscoelastic parameters of the asphalt material.

According to the time-temperature superposition principle [16,17], 25 °C was selected as the reference temperature. The complex shear modulus measured at 15 °C, 20 °C, 30 °C, and 35 °C was shifted to align with the master curve of the complex shear modulus of the asphalt. The shift factors at each temperature were quantitatively determined under the reference temperature conditions, and the reduced angular frequency coordinates for the master curve were calculated, as shown in Equation (1):

(1) $$\log\omega_{r}=\log\varphi_{T}+\log\omega$$

where ω is angular frequency in radians per second (rad/s); $\omega_{r}$ is reduced angular frequency in radians per second (rad/s); and $\varphi_{T}$ is the shift factor.

#### 2.3.2. Linear Amplitude Sweep (LAS) Test

**Figure 3:**
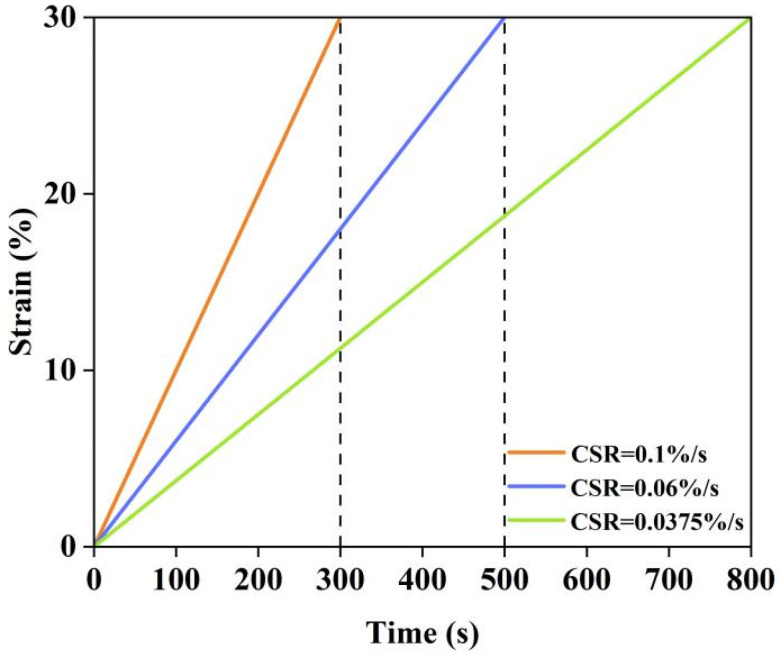
LAS test loading program.

Linear Amplitude Sweep (LAS) tests were performed using a dynamic shear rheometer [18] in controlled strain mode. The strain was applied with a loading frequency of 10 Hz, and the amplitude of the sinusoidal load on the asphalt material was linearly increased from 0.1% to 30% at a test temperature of 25 °C, with a standard loading time of 300 s. For this section, a fatigue life prediction method based on the fatigue failure criterion was employed. LAS tests under various loading conditions were conducted to fit the virtual strain energy release rate and the number of cyclic loadings, obtaining the relevant parameters for calculating fatigue life. Consequently, LAS tests with scan times of 300 s, 500 s, and 800 s were performed on the modified bitumen specimens. The LAS test loading procedure utilized in this section is illustrated in Figure 3.

This study analyzes the fatigue performance of modified asphalt using the Simplified Viscoelastic Continuum Damage (S-VECD) theory [19]. The S-VECD theory is not constrained by specific test conditions and defines the change in the internal state of the material during loading as the damage variable. It establishes the relationship between the change in the damage variable S and time t, allowing for a quantitative analysis of the fatigue damage behavior of the material. The peak virtual strain during the loading cycle of modified asphalt is expressed in Equation (2) [20]:

(2) $$\gamma_{P\;}^{R}=\frac{1}{G_{R}}\cdot \left({\left|G^{*}\right|}_{\mathrm{LVE}}\cdot \gamma_{P}\right)$$

where $\gamma_{P}$ is the virtual strain; $G_{R}$ is the reference modulus of the material, taken as 1 MPa in this paper; and ${\left|G^{*}\right|}_{\mathrm{LVE}}$ is the dynamic shear modulus of the asphalt material in linear viscoelasticity.

The relationship between the complex modulus C and the internal damage variable S of the asphalt material is expressed by Equation (3):

(3) $$C\left(S\right)=\frac{\tau_{P}}{\gamma_{P}^{R}\cdot \mathrm{DMR}}$$

where $\tau_{P}$ is the peak shear stress of the asphalt material.

The relationship between the damage variable of the asphalt material and time during the loading process is described by Equation (4):

(4) $$S\left(t\right)=\sum_{i=1}^{N}{\left[\frac{\mathrm{DMR}}{2}{\left(\gamma_{P}^{R}\right)}^{2}\left(C_{i-1\;}-C_{i}\right)\right]}^{\frac{\alpha }{1+\alpha }}\times \;{\left(t_{\mathrm{Ri}\;}-{\;t}_{\mathrm{Ri}-1}\right)}^{\frac{1}{1+\alpha }}$$

(5) $$t_{\mathrm{Ri}}=\frac{t_{i}}{\varphi_{T}}$$

where DMR is the ratio of the initial dynamic shear modulus of the asphalt material to the dynamic shear modulus of the asphalt material in linear viscoelasticity; i is the loading time; and $\varphi_{T}$ is the shift factor.

Based on the experimental data, the complex modulus C and damage variable S are fitted to derive the C-S curve of the material. The fitting formula is given in Equation (6):

(6) $$C\left(t\right)=1\;-C_{1}\cdot S{\left(t\right)}^{C_{2}}$$

where $C_{1}$ and $C_{2}$ are the fitting parameters of the material. 

In the context of the Simple Viscoelastic Continuum Damage (S-VECD) theory model, the maximum stored pseudo-strain energy (PSE) is defined as the fatigue failure point of the asphalt material. The formulas for calculating the stored energy, total pseudo-strain energy, and released energy are given in Equations (7), (8), and (9), respectively:

(7) $$W_{s}^{R}=\frac{1}{2}\;\times \;\tau_{p}\;\times \;\frac{\gamma_{p}^{R}}{\mathrm{DMR}}=\frac{1}{2}\;\times \;C\;\times {\;\left(\gamma_{p}^{R}\right)}^{2}$$

(8) $$W_{t}^{R}=\frac{1}{2}\;\times \;\tau_{\mathrm{undamage}}\;\times \;\gamma_{p}^{R\;}=\frac{1}{2}\;\times \;{\left(\gamma_{p}^{R}\right)}^{2}$$

(9) $$W_{r}^{R}={\;W}_{t}^{R}-{\;W}_{s}^{R}=\frac{1}{2}\;\times \;\left(1-\;C\right){\left(\gamma_{p}^{R}\right)}^{2}$$

**Figure 4:**
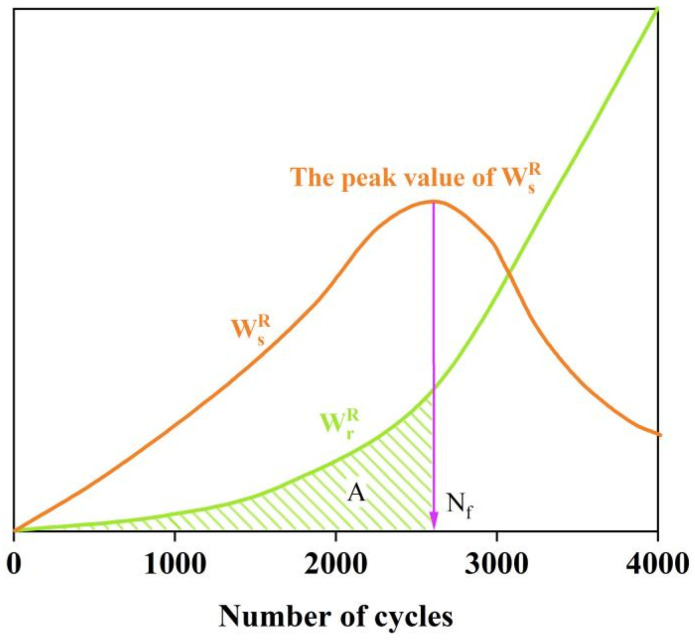
Evolution of stored energy and released energy in LAS test.

The relationship between the stored energy and the released energy during the LAS test loading process is illustrated in Figure 4.

Zhang proposed a fatigue failure criterion based on the virtual strain energy release rate ($G^{R}$), stating that the relationship between $G^{R}$ and fatigue life ($N_{f}$) is an inherent property of the material [21]. Therefore, this study predicts the fatigue life of modified asphalt based on the virtual strain energy release rate ($G^{R}$) [22,23]. The virtual strain energy release rate ($G^{R}$) refers to the average release rate of pseudo-strain energy (PSE) per cycle before fatigue failure occurs during the fatigue test process. It is necessary to first calculate the average release of PSE per cycle from the beginning of the test until fatigue failure is reached, denoted as $\bar{W_{r}^{R}}$, and then Equation (10) can be used to calculate the virtual strain energy release rate ($G^{R}$).

(10) $$G^{R}=\frac{\bar{W_{r}^{R}}}{N_{f}}=\frac{\frac{A}{N_{f}}}{N_{f}}=\frac{A}{{\left(N_{f}\right)}^{2}}$$

where A is the area under the curve of the released pseudo-strain energy before the material reaches fatigue failure, as indicated by the shaded area in Figure 4. $N_{f}$ denotes the number of loading cycles. 

There is a strong correlation between the virtual strain energy release rate ($G^{R}$) and the number of loading cycles ($N_{f}$) under logarithmic coordinates, which can be fitted using Equation (11):

(11) $$G^{R}=a{N_{f}}^{b}$$

where a and b are fitting parameters.

The fatigue life prediction equation for asphalt, based on the fatigue failure criterion, is presented in Equation (12):

(12) $$N_{f}={\left[\frac{K}{a}\;\times \;{\left(\gamma \right)}^{2+2\alpha \left(\frac{C_{2}}{Q}\right)}\right]}^{\frac{1}{b+1-\frac{C_{2}}{Q}}}$$

where K and Q are parameter combination values, which can be calculated using Equations (13)–(15):

(13) $$K=\frac{1}{2}\;\times \;C_{1\;}\times \;{\left({\left|G^{*}\right|}_{\mathrm{LVE}}\right)}^{2}\;\times \;P^{\left(-\frac{C_{2}}{Q}\right)}\;\times \;\frac{1}{\left(\frac{C_{2}}{Q}\right)+1}$$

(14) $$Q=1\;-\;\alpha \;\times {\;C}_{2}+\alpha$$

(15) $$P=\frac{f\;\times \;2^{\alpha }}{Q{\left(C_{1\;}\times \;C_{2}\right)}^{\alpha }{\left({\left|G^{*}\right|}_{\mathrm{LVE}}\right)}^{2\alpha }}$$

Equation (12) can be utilized to predict the fatigue life of asphalt materials under varying strains, thus offering a comprehensive analysis of their fatigue performance.

#### 2.3.3. Fatigue–Healing–Fatigue Tests

**Figure 5:**
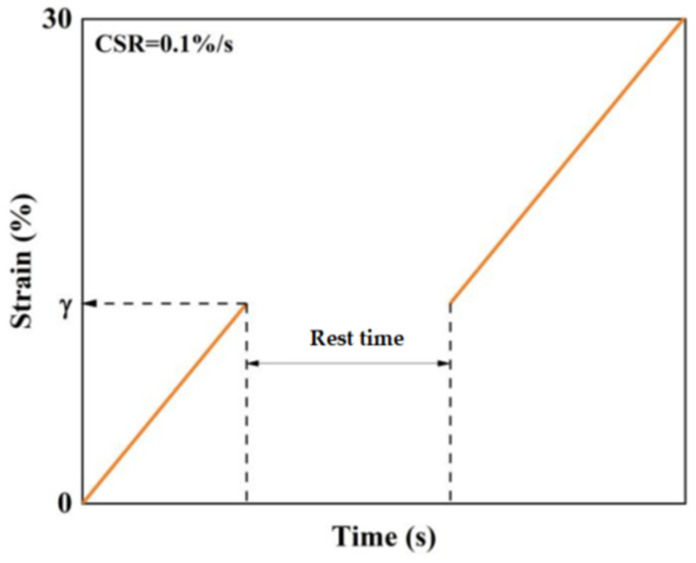
Loading procedure for LAS-based self-healing test.

In this study, an interval period was introduced to the standard continuous LAS test to evaluate the self-healing performance of asphalt binders by calculating the change in the damage variable S before and after the interval. The damage parameter D corresponding to the peak pseudo-strain energy (PSE) of the standard continuous LAS test was defined as the damage parameter at fatigue failure of the modified asphalt. The strain level corresponding to a 50% damage degree was selected as the strain level before the onset of healing. The LAS test was conducted on the asphalt specimens at a standard rate and temperature of 25 °C. Loading was halted when the strain reached the selected level, and the specimens were left to stand for 1800 s. The LAS loading was then resumed until the strain reached 30%. The strain amplitude at the start of the second loading stage was the same as at the end of the first, and the loading rate remained consistent across both stages. The loading procedure is illustrated in Figure 5.

**Figure 6:**
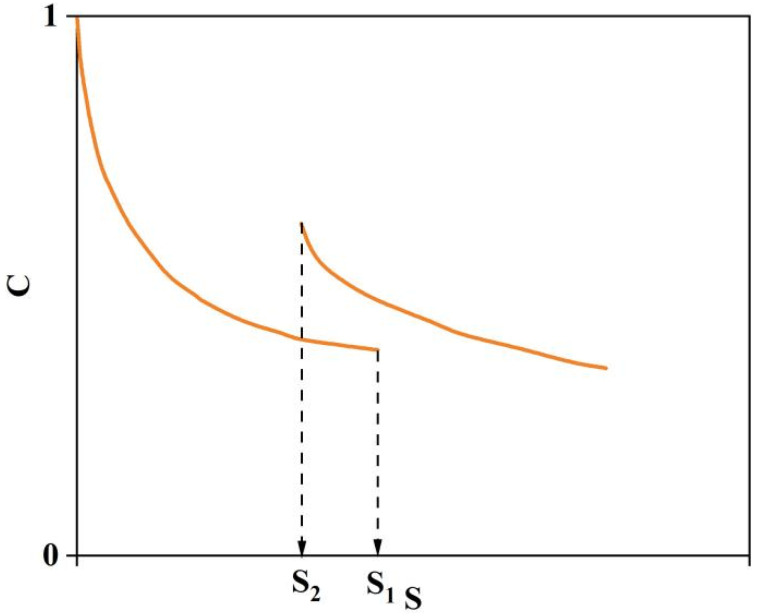
Damage characteristic curve of LASH test.

In this study, the damage healing percentage $\%H_{S}$ was selected as the self-healing indicator, calculated according to Equation (16), The damage characteristic curve of Lash test is shown in Figure 6:

(16) $$\%H_{S}=\frac{S_{1}-S_{2}}{S_{1}}\;\times \;100\%$$

where $S_{1}$ and $S_{2}$ are the damage variables S of the asphalt material before and after the rest period, respectively. It is important to note that when calculating $S_{2}$, the time t needs to be subtracted by the duration of the rest period [24]. 

## 3. Results and Discussion

### 3.1. Line Viscoelastic Analysis

**Figure 7:**
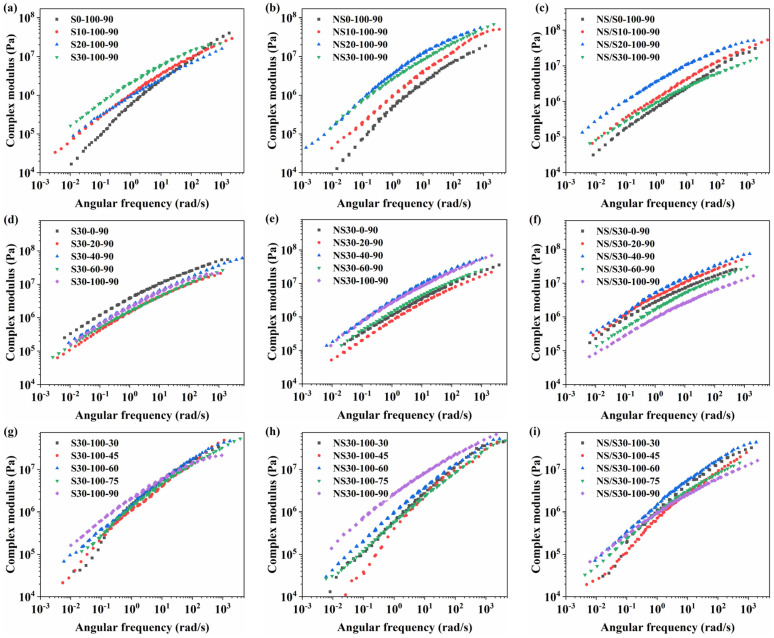
Main curves of complex modulus: (**a**,**d**,**g**) represent SBS-modified asphalt; (**b**,**e**,**h**) represent nano-SiO_2_-modified asphalt; (**c**,**f**,**i**) represent nano-SiO_2_/SBS composite modified asphalt.

The complex modulus $G^{*}$ is a crucial parameter that describes the viscoelastic behavior of asphalt materials under dynamic loading conditions. It is positively correlated with the reduced angular frequency and can be used to characterize the material’s ability to resist deformation. A higher value of $G^{*}$ indicates a greater resistance to deformation and is a crucial parameter that describes the viscoelastic behavior of asphalt materials under dynamic loading conditions. The influence of aging time, wet–dry cycles, and aging temperature on the trend in the main curves of the complex modulus for SBS-modified asphalt, nano-SiO_2_-modified asphalt, and nano-SiO_2_/SBS composite modified asphalt is illustrated in Figure 7.

Figure 7 shows that the complex modulus of SBS-modified asphalt, nano-SiO_2_-modified asphalt, and nano-SiO_2_/SBS composite modified asphalt gradually increases with prolonged aging time, indicating an overall increase in stiffness and improved resistance to deformation. As aging time extends, the modified asphalt becomes harder and more brittle in the high-frequency, low-temperature region, leading to decreased low-temperature cracking resistance and increased susceptibility to cracking. In the low-frequency, high-temperature region, the complex modulus of the modified asphalt increased, indicating enhanced resistance to deformation. The effect of aging time on the low-temperature performance of SBS-modified asphalt was less significant compared to that of nano-SiO_2_-modified asphalt and nano-SiO_2_/SBS composite modified asphalt. During shorter aging periods, nano-SiO_2_-modified asphalt and nano-SiO_2_/SBS composite modified asphalt exhibited better low-temperature cracking resistance, suggesting that the addition of nano-SiO_2_ powder improves the low-temperature performance of asphalt materials. However, this advantage diminished with increasing aging time.

The influence of different wet–dry conditions on the complex modulus of various modified asphalt types was diverse. The alternating cycles of water vapor and oxygen decreased the complex modulus of SBS-modified asphalt across the entire frequency range, leading to reduced deformation resistance. This occurs because the butadiene segments in the SBS modifier are susceptible to decomposition in the presence of moisture and oxygen, causing the network structure in SBS-modified asphalt to break down [25]. In contrast, the complex modulus of nano-SiO_2_-modified asphalt and nano-SiO_2_/SBS composite modified asphalt exhibited an increasing-then-decreasing trend. Under the condition of a 40% water content–time ratio, they exhibited higher stiffness, making them more susceptible to cracking at low temperatures while demonstrating good resistance to deformation at high temperatures. Additionally, the complex modulus of nano-SiO_2_/SBS composite modified asphalt varied significantly with increasing water content–time ratio, indicating its sensitivity to changes in the external wet–dry environment.

As aging temperature increased, SBS-modified asphalt and nano-SiO_2_-modified asphalt exhibited varying degrees of increase in complex modulus across the entire frequency range. This phenomenon occurs because the rise in temperature accelerates the movement of water vapor molecules, facilitating reactions between these molecules and the active components in the asphalt, leading to asphalt hardening and increased stiffness. Additionally, the increased temperature accelerates the volatilization of light components in the asphalt. The complex modulus of nano-SiO_2_-modified asphalt exhibited significant variations with increasing temperature, and the slope of the complex modulus master curve was steep, indicating a high sensitivity of nano-SiO_2_-modified asphalt to temperature changes. Moreover, the increase in temperature enhanced the high-temperature deformation resistance and low-temperature crack resistance of nano-SiO_2_/SBS composite modified asphalt, although the specific mechanisms require further investigation.

In general, the extension of aging time and the influence of wetting and drying cycles enhance the temperature sensitivity of SBS-modified asphalt, nano-SiO_2_-modified asphalt, and nano-SiO_2_/SBS composite modified asphalt. An increase in aging temperature decreases the temperature sensitivity of modified asphalt. In addition, SBS-modified asphalt and nano-SiO_2_/SBS composite modified asphalt exhibit low sensitivity to temperature changes. With the increase in the proportion of water content, the resistance to deformation of SBS-modified asphalt decreases, while the resistance to deformation of nano-SiO_2_-modified asphalt and nano-SiO_2_/SBS composite modified asphalt shows a trend of first increasing and then decreasing. The increase in aging temperature improves the overall deformation resistance of SBS-modified asphalt and nano-SiO_2_-modified asphalt and also improves the high-temperature performance and low-temperature crack resistance of nano-SiO_2_/SBS composite modified asphalt.

### 3.2. Fatigue Performance Analysis

**Figure 8:**
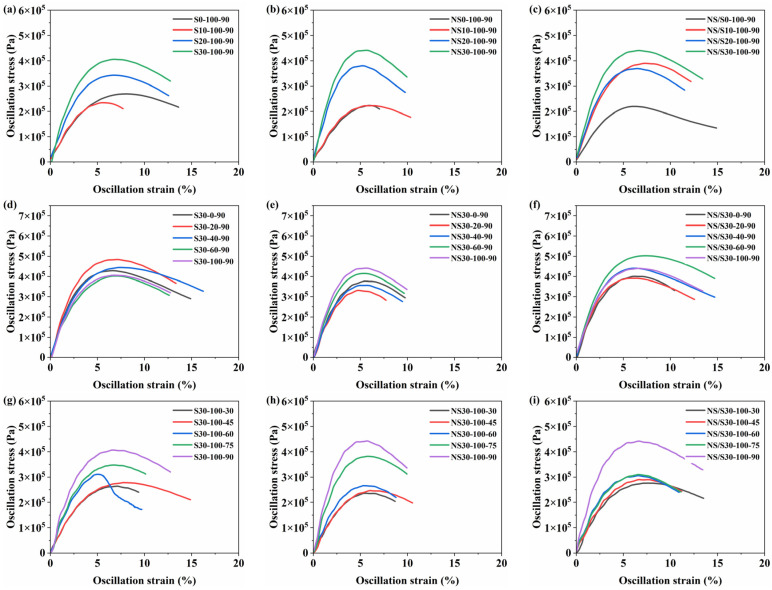
Stress–Strain relationship graph: (**a**,**d**,**g**) represent SBS-modified asphalt; (**b**,**e**,**h**) represent nano-SiO_2_-modified asphalt; (**c**,**f**,**i**) represent nano-SiO_2_/SBS composite modified asphalt.

Within the specified strain range, the shear stress of the asphalt material increases with increasing shear strain, reaches a peak, and then begins to decrease, indicating the occurrence of fatigue damage in the material. After exposure to moisture, the peak stress and peak strain of the modified asphalt materials exhibit varying degrees of change. The influence of aging time, dry–wet conditions, and aging temperature on the stress–strain relationship of SBS-modified asphalt, nano-SiO_2_-modified asphalt, and nano-SiO_2_/SBS composite modified asphalt is illustrated in Figure 8.

Figure 8 shows that as aging time increased, the peak stress of modified asphalt increases while the peak strain decreased. Additionally, the width of the peak stress plateau narrowed, indicating that increased aging time reduced the stress–strain bearing capacity of the modified asphalt during loading. Among the three types of modified asphalt, the nano-SiO_2_-modified asphalt had the narrowest peak stress plateau and reached the fatigue failure point earliest, indicating the poorest deformation resistance. Conversely, the nano-SiO_2_/SBS composite modified asphalt exhibited a wider peak stress plateau and a later occurrence of the fatigue failure point, indicating its better resistance to deformation damage.

As the percentage of moisture content increased, the peak stress of the three types of modified asphalt initially increased and then decreased. Under conditions of 20%, 100%, and 60% moisture content, SBS-modified asphalt, nano-SiO_2_-modified asphalt, and nano-SiO_2_/SBS composite modified asphalt exhibited the highest peak stress, respectively, accompanied by a decrease in strain sensitivity.

As the aging temperature increased, the peak stress of the modified asphalt material increased while the peak strain decreased. This indicates that the increased aging temperature reduced the strain dependency of the modified asphalt and accelerated its aging process. Additionally, the increase in aging temperature narrowed the width of the peak stress plateau, reducing the shear deformation that the modified asphalt can withstand, indicating a deterioration in its fatigue performance. It can be found that the extension of aging time, the alternation of dry and wet environments, and the increase in aging temperature all reduce the strain sensitivity of modified asphalt. The nano-SiO_2_/SBS composite modified asphalt has the best resistance to deformation, followed by SBS-modified asphalt, and the nano-SiO_2_-modified asphalt has the worst resistance to deformation.

**Figure 9:**
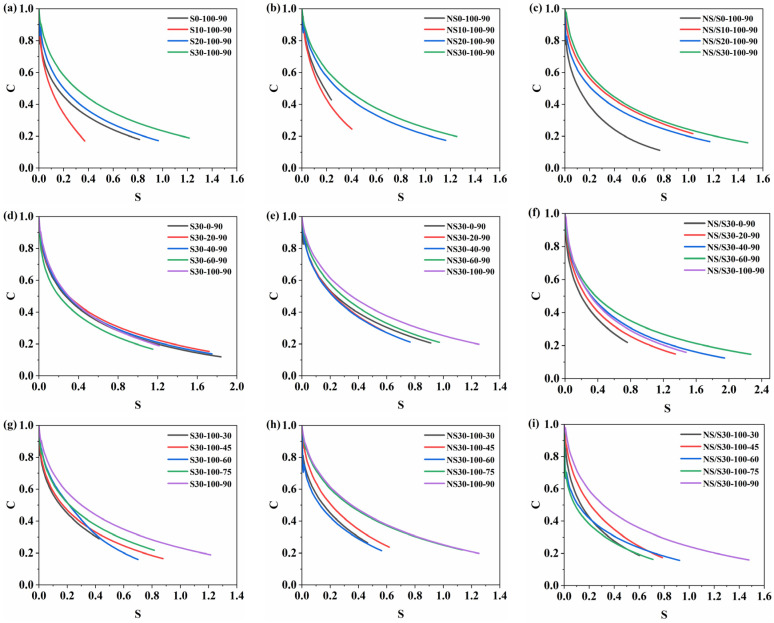
C-S curve graph: (**a**,**d**,**g**) represent SBS-modified asphalt; (**b**,**e**,**h**) represent nano-SiO_2_-modified asphalt; (**c**,**f**,**i**) represent nano-SiO_2_/SBS composite modified asphalt.

When the asphalt material is in an undamaged state, the storage modulus C = 1 and the damage variable S=O. As the value of C gradually decreases and S increases, the asphalt material evolves from an undamaged state to a fatigue failure state. The rate of decline in the curve gradually decreases, indicating a reduced sensitivity of the asphalt material to the load. The influence of aging time, wet–dry conditions, and aging temperature on the C-S curves of SBS-modified asphalt, nano-SiO_2_-modified asphalt, and nano-SiO_2_/SBS composite modified asphalt is shown in Figure 9, where the endpoint of the fatigue damage curve is defined based on the peak value of stored virtual strain energy.

Figure 9 shows that as aging time increases, the damage variable corresponding to the fatigue failure point of the modified asphalt also increases. This indicates that prolonged aging increases the cumulative damage variable, leading to fatigue failure. At the same damage level, the increase in aging time enhanced the structural integrity of the modified asphalt, resulting in a slower decline in pseudo-stiffness. Compared to SBS-modified asphalt and nano-SiO_2_-modified asphalt, the value of S at the fatigue failure point of nano-SiO_2_/SBS composite modified asphalt was larger, indicating that nano-SiO_2_/SBS composite modified asphalt accumulated the most damage at the state of fatigue damage.

The presence of moisture reduced the value of S at which SBS-modified asphalt experienced fatigue failure, resulting in decreased cumulative damage. The reduction in S value is most pronounced under conditions of 60% and 100% moisture content, indicating poor resistance of SBS-modified asphalt to moisture erosion and a significant weakening of its fatigue resistance under high moisture content conditions. For nano-SiO_2_-modified asphalt, under conditions of 60% and 100% moisture content, the C-S curve is above the C-S curve under dry conditions, indicating an increase in S value. Conversely, under conditions of 20% and 40% moisture content, the C-S curve is below the C-S curve under dry conditions, indicating a decrease in S value. This suggests that at the same damage level, higher moisture content increased the structural integrity of nano-SiO_2_-modified asphalt, while lower moisture content reduced it. Nano-SiO_2_-modified asphalt exhibited the best fatigue resistance under 100% moisture content conditions. For nano-SiO_2_/SBS composite modified asphalt, the increase in moisture content delayed the occurrence of fatigue failure to varying degrees. Under conditions of 60% moisture content, nano-SiO_2_/SBS composite modified asphalt could withstand the most cumulative damage.

The increase in aging temperature led to an increase in the cumulative damage at which asphalt samples experienced fatigue failure. This is due to the fact that higher aging temperatures accelerate the volatilization of light components in the asphalt and increase the content of polar groups. Consequently, the characteristic damage curve of the modified asphalt at higher aging temperatures becomes smoother, with higher cumulative damage.

Summarizing the influence of three factors, the increase in aging time and the increase in aging temperature increase the cumulative damage value of modified asphalt. The increase in the proportion of water-bearing time weakens the ability of SBS-modified asphalt to resist fatigue damage, but overall enhances the fatigue performance of modified asphalt containing nano-SiO_2_ powder.

**Figure 10:**
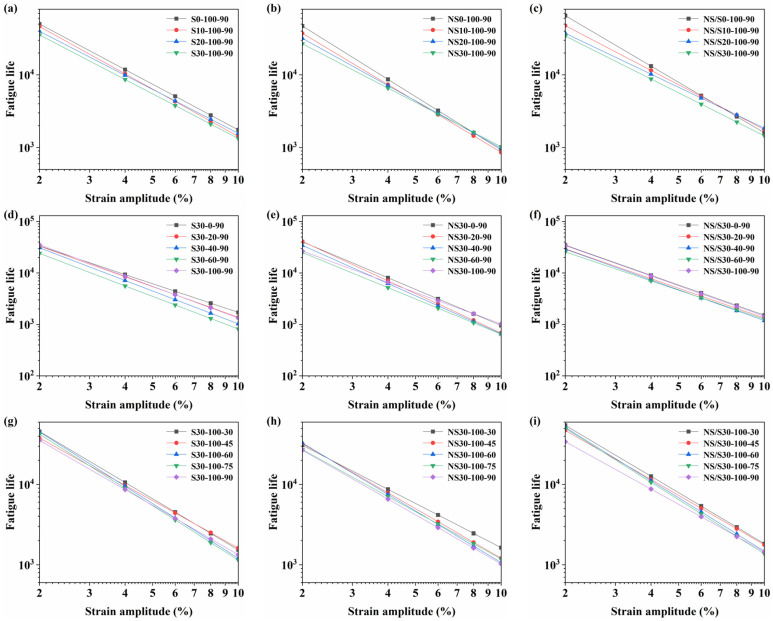
Fatigue life prediction curve: (**a**,**d**,**g**) represent SBS-modified asphalt; (**b**,**e**,**h**) represent nano-SiO_2_-modified asphalt; (**c**,**f**,**i**) represent nano-SiO_2_/SBS composite modified asphalt.

Using Equation (12), the fatigue life of asphalt material can be predicted under various strain levels. This study analyzed the fatigue life of modified asphalt at strain levels of 2%, 4%, 6%, 8%, and 10%, providing a comprehensive analysis of the material’s fatigue performance. The strain sensitivity of modified asphalt is indicated by the slope of the fatigue life prediction curve in double logarithmic coordinates. A greater slope indicates higher sensitivity of the fatigue life of modified asphalt to strain changes. The fatigue life prediction curves are illustrated in Figure 10.

Figure 10 shows that with increasing aging time, the fatigue life of modified asphalt exhibited a decreasing trend. The influence of aging time on the fatigue life of modified asphalt was not significant under low strain conditions. However, under high strain conditions, there was a noticeable divergence in the fatigue life of the same type of modified asphalt. In a water environment, compared to nano-SiO_2_-modified asphalt, SBS-modified asphalt and nano-SiO_2_/SBS composite modified asphalt exhibited higher fatigue life under the same strain conditions, indicating their ability to withstand more repetitions of loading cycles. The fatigue life prediction curves of the three types of modified asphalt showed varying degrees of counterclockwise rotation with increasing aging time, with SBS-modified asphalt exhibiting a less pronounced rotation, suggesting relatively stable strain sensitivity. Additionally, the slope of the fatigue life prediction curve for nano-SiO_2_-modified asphalt was larger than that of the other two types, indicating that nano-SiO_2_-modified asphalt was most sensitive to strain variations.

Under dry and 100% water content conditions, modified asphalt exhibited relatively higher fatigue life, while alternating wet and dry environments reduced the fatigue life of modified asphalt. The fatigue life of modified asphalt decreased with an increase in water content percentage. SBS-modified asphalt demonstrated a longer fatigue life under dry and 20% water content conditions, whereas nano-SiO_2_/SBS composite modified asphalt exhibited a longer fatigue life under 40%, 60%, and 100% water content conditions. Nano-SiO_2_-modified asphalt had the lowest fatigue life.

An increase in aging temperature in water environments reduced the fatigue life of modified asphalt, with nano-SiO_2_/SBS composite modified asphalt experiencing a smaller decrease in fatigue life. This indicates that nano-SiO_2_/SBS composite modified asphalt is less sensitive to changes in aging temperature. Additionally, under the same aging temperature, modified asphalt exhibited a phenomenon where fatigue life was lower at low strains and higher at high strains. This may be related to the nonlinear viscoelastic properties of asphalt, although the specific reasons require further investigation [25].

**Figure 11:**
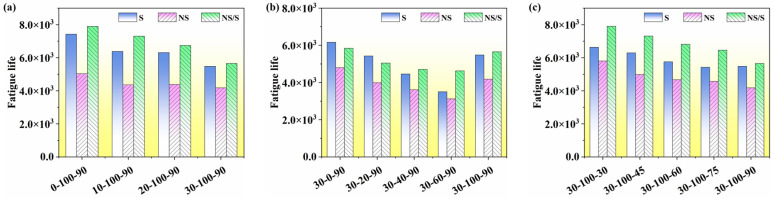
Fatigue life of modified asphalt at 5% strain level: (**a**) SBS-modified asphalt, (**b**) nano-SiO_2_-modified asphalt, (**c**) nano-SiO_2_/SBS composite modified asphalt.

As shown in Figure 11, under the influence of a water environment, the fatigue life of nano-SiO_2_-modified asphalt is the lowest among the three types of modified asphalt. In contrast, SBS-modified asphalt and nano-SiO_2_/SBS composite modified asphalt exhibit higher fatigue life under different conditions of water exposure. Increased aging time and aging temperature reduce the fatigue life of modified asphalt in the presence of water, with nano-SiO_2_/SBS composite modified asphalt demonstrating the highest fatigue life. Modified asphalt has the highest fatigue life in dry environments, followed by conditions with 100% water content time share. The fatigue life of modified asphalt in alternating wet and dry environments decreases significantly, as evidenced by the reduction in fatigue life with increased water content time.

### 3.3. Self-Healing Performance Analysis

**Figure 12:**
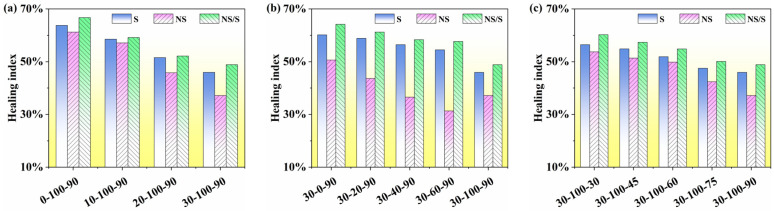
Self-healing index: (**a**) SBS-modified asphalt, (**b**) nano-SiO_2_-modified asphalt, (**c**) nano-SiO_2_/SBS composite modified asphalt.

Based on the S-VECD theory and Equation (16), an analysis of the self-healing index of modified asphalt under a water environment is conducted. The self-healing index illustrates the relationship between pseudo-stiffness and damage strength, where a higher index indicates stronger self-healing capability of the material. The self-healing indices of SBS-modified asphalt, nano-SiO_2_-modified asphalt, and nano-SiO_2_/SBS composite modified asphalt under the influence of aging time, wet–dry conditions, and aging temperature are illustrated in Figure 12.

Figure 12 shows that SBS-modified asphalt and nano-SiO_2_/SBS composite modified asphalt exhibited relatively high self-healing indices. This is attributed to the elastic recovery effect exerted by the polybutadiene segments of the SBS polymer. The self-healing capability of nano-SiO_2_/SBS composite modified asphalt was even stronger. This is because during the oxidation process of asphalt, carbonyl groups are generated, which further react with water molecules to produce carboxylic acids, enhancing the hydrophilicity of modified asphalt [26,27]. This phenomenon leads to the hardening of the structural film at the asphalt–water interface, preventing self-healing behavior in the modified asphalt. However, the hydrophobic nature of nano-SiO_2_ particles can mitigate this phenomenon to some extent. Additionally, the incorporation of nano-SiO_2_ contributes to the stabilization of the SBS polymer structure, thereby enhancing the self-healing capability of modified asphalt. The extension of aging time, increase in water content, and elevation of aging temperature all resulted in a decrease in the self-healing index of modified asphalt, indicating a deterioration in self-healing ability. This occurs because, with increased aging, the content of macromolecules in modified asphalt increases, leading to a degradation in self-healing ability and a decrease in the self-healing index. SBS-modified asphalt and nano-SiO_2_/SBS composite modified asphalt have high self-healing abilities, and the self-healing ability of nano-SiO_2_/SBS composite modified asphalt is the best. In addition, all three forms of water action hinder the occurrence of self-healing behavior in modified asphalt.

**Figure 13:**
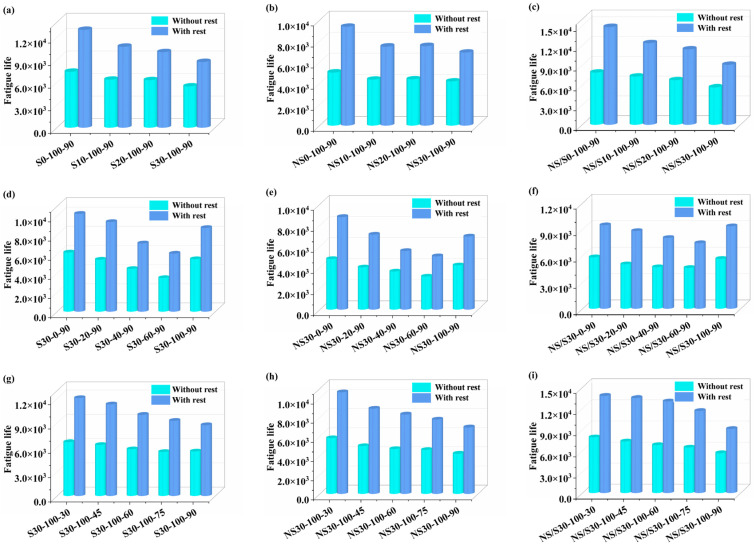
Comparison of fatigue life before and after healing: (**a**,**d**,**g**) represent SBS-modified asphalt; (**b**,**e**,**h**) represent nano-SiO_2_-modified asphalt; (**c**,**f**,**i**) represent nano-SiO_2_/SBS composite modified asphalt.

The self-healing property of asphalt material is a major factor causing differences between predicted fatigue life and actual life. The fatigue life before and after self-healing of modified asphalt at a shear strain of 5% was calculated using the S-VECD theory and Equation (12), and the calculation results are shown in Figure 13.

Figure 13 shows that a significant increase in the fatigue life of the modified asphalt occurs after an interval of 1800 s, indicating that the self-healing compensatory behavior of the asphalt material improves fatigue life. This improvement is related to the restoration of the asphalt modulus during the interval. In the presence of moisture, the fatigue life of nano-SiO_2_/SBS composite modified asphalt was higher than that of SBS-modified asphalt and nano-SiO_2_-modified asphalt, with nano-SiO_2_-modified asphalt having the lowest fatigue life. When considering self-healing compensation behavior, the fatigue life of the three modified asphalts showed different rates of decrease with the increase in aging time and aging temperature. This was attributed to the increase in the hard component of the modified asphalt during the aging process, which made the asphalt hard and brittle, reducing its fatigue life. As the proportion of water content time increased, the fatigue life of modified asphalt initially decreased and then increased, with the highest fatigue life occurring in dry environments. The fatigue life of modified asphalt in alternating wet and dry environments was significantly reduced. Comparing the fatigue life of the three modified asphalts in dry and alternating wet and dry environments, it was found that the fatigue life of nano-SiO_2_/SBS composite modified asphalt was higher under the effects of 40%, 60%, and 100% water content time share. In contrast, SBS-modified asphalt had a higher fatigue life in dry environments and under the effect of 20% water content time share.

## 4. Conclusions

This study conducted tests on modified asphalt samples using a dynamic shear rheometer and processed the measured data based on the simplified viscoelastic continuous damage theory to investigate the viscoelastic properties, fatigue performance, and self-healing properties of modified asphalt under the influence of aging time, dry–wet conditions, and aging temperature. The main conclusions from the research are as follows:

(1) With the extension of aging time, the overall stiffness of modified asphalt increased, which enhanced the high-temperature performance but deteriorated the low-temperature crack resistance. The addition of nano-SiO_2_ powder improved the low-temperature performance of the asphalt material. Under alternating dry–wet conditions, the deformability of SBS-modified asphalt decreased. However, nano-SiO_2_-modified asphalt and nano-SiO_2_/SBS composite modified asphalt exhibited good resistance to external loads under 40% moisture content conditions. Increasing aging temperature improved the high-temperature performance and low-temperature crack resistance of nano-SiO_2_/SBS composite modified asphalt.
(2) The extension of aging time, alternating dry–wet environments, and the increase in aging temperature all reduced the strain sensitivity of modified asphalt. Nano-SiO_2_/SBS composite modified asphalt had the best deformation resistance, which is about 30% higher than SBS-modified asphalt, while nano-SiO_2_-modified asphalt had the poorest deformation resistance.
(3) SBS-modified asphalt had a longer fatigue life under dry conditions and 20% moisture content conditions. Nano-SiO_2_/SBS composite modified asphalt withstood more load cycles under four aging times (0 h, 10 h, 20 h, and 30 h), five aging temperatures (30 °C, 45 °C, 60 °C, 75 °C, and 90 °C), and three moisture content conditions (40%, 60%, and 100%) in water environments.
(4) Modified asphalt considering self-healing compensation behavior exhibited longer fatigue life. The extension of aging time, increase in moisture content, and rise in aging temperature all reduced the self-healing ability and fatigue life after self-healing of modified asphalt. The self-healing index of nano-SiO_2_/SBS composite modified asphalt is greater than 50%, indicating the best self-healing ability.

This article conducted water environment aging tests on modified asphalt under high temperature conditions but we have not yet studied the influence of water environment on the performance of modified asphalt under low-temperature conditions. In order to further improve the research, low-temperature water environment tests can be designed in the future to deeply explore the changes in the fatigue self-healing performance of modified asphalt under low-temperature water environment conditions.

## Figures and Tables

**Table 1:** Basic performance indicators of 70# road petroleum asphalt.

Technical Specifications	Test Value	Technical Requirements	Unit	Test Method
Penetration depth (25 °C, 100 g, 5 s)	72.8	60–80	0.1 mm	T0604-2011
Softening point	50.6	≥46	°C	T0606-2011
Ductility (15 °C)	>100	≥100	cm	T0605-2011

**Table 2:** Main technical parameters of SBS modifier.

Technical Indicators	Measured Value	Unit
Oil content	0.68	%
Total ash content	0.21	≤%
Volatile matter	1.05	≤%
S/B Block ratio	30/70	-
Tensile strength	18.0	≥MPa

**Table 3:** Main technical parameters of hydrophobic nano-SiO_2_ powder.

Technical Indicators	Measured Value	Unit
Purity	≥99.9	%
Chemical properties	Oil-loving hydrophobic	-
Average particle size	20	nm
Specific surface area	190	m^2^/g
Bulk density	0.15	g/cm^3^

**Table 4:** Asphalt samples and test conditions.

Influencing Factors	Asphalt Samples	Test Conditions			Number
		Time (h)	Proportion of Time with Water (%)	Temperature (°C)	
Aging time	Nano-SiO_2_/SBS composite modified asphalt Nano-SiO_2_ modified asphalt, SBS modified asphalt,	0	100	90	S/NS/(NS/S)0-100-90
		10			S/NS/(NS/S)10-100-90
		20			S/NS/(NS/S)20-100-90
		30			S/NS/(NS/S)30-100-90
Dry-wet conditions		30	0	90	S/NS/(NS/S)30-0-90
			20		S/NS/(NS/S)30-20-90
			40		S/NS/(NS/S)30-40-90
			60		S/NS/(NS/S)30-60-90
			100		S/NS/(NS/S)30-100-90
Aging temperature		30	100	30	S/NS/(NS/S)30-100-30
				45	S/NS/(NS/S)30-100-45
				60	S/NS/(NS/S)30-100-60
				75	S/NS/(NS/S)30-100-75
				90	S/NS/(NS/S)30-100-90

## Data Availability

The original contributions presented in the study are included in the article; further inquiries can be directed to the corresponding authors.
